# Computer-Guided Surface Engineering for Enzyme Improvement

**DOI:** 10.1038/s41598-018-30434-5

**Published:** 2018-08-10

**Authors:** Matthew Wilding, Colin Scott, Andrew C. Warden

**Affiliations:** 1 CSIRO Land & Water, Black Mountain Laboratories, Canberra, ACT 2601 Australia; 2 Research School of Chemistry, Australian National University, Building 137 Sullivan’s Creek Road, Canberra, ACT 2601 Australia

## Abstract

Protein engineering strategies are often guided by our understanding of how the structure of a protein determines its function. However, our understanding is generally restricted to small regions of a protein, namely the active site and its immediate vicinity, while the remainder of the protein is something of an enigma. Studying highly homologous transaminases with strictly conserved active sites, but different substrate preferences and activities, we predict and experimentally validate that the surface of the protein far from the active site carries out a decisive role in substrate selectivity and catalytic efficiency. Using a unique molecular dynamics approach and novel trajectory analysis, we demonstrate the phenomenon of surface-directed ligand diffusion in this well-known protein family for the first time. Further, we identify the residues involved in directing substrate, design surface channel variants endowed for improved kinetic properties and establish a broadly applicable new approach for protein engineering.

## Introduction

Our ability to engineer proteins has been paramount for their implementation in a variety of applications. In biocatalysis, the necessity to expand and alter substrate range, improve kinetic parameters or physiochemical properties has driven the development of a range of engineering strategies. However, for all the advances in the field, our understanding of how proteins fundamentally function, or interact with their substrates is still limited. Furthermore, most reported engineering approaches to improve catalysis fall into one of two categories. The first is rational design approaches, which sample small, focussed libraries. However, they usually concentrate on the active sites of enzymes, the “catalytic centers”^1^. As such, the contribution the rest of the protein makes to catalysis remains unknown and under-investigated. Alternatively, random mutagenesis samples the whole protein, but typically requires an accompanying high-throughput analytical method in order to achieve sufficient sampling, which depending on the aim of the investigation, is not always feasible. It often identifies residues far-removed from the active site, but rationalising a causal relationship is often difficult, and ultimately, regardless of the approach adopted, a large portion of most proteins remains uncharted territory. However, there are already examples in the literature suggesting that surface residues, far-removed from the active site, may contribute to catalytic efficiency in proteins, although the tools to identify and validate these findings remain limited, and canonical molecular dynamics approaches have until now typically been considered impractical^2^.

**Figure 1:**
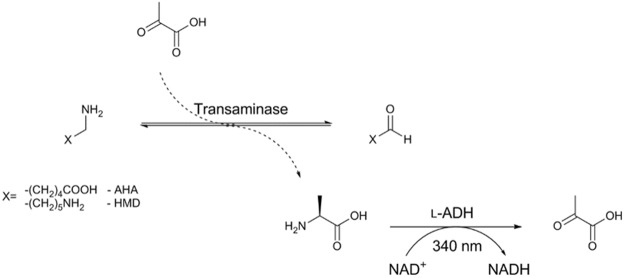
Illustration of the alanine dehydrogenase coupled assay used in screening. L-ADH – alanine dehydrogenase.

We recently reported a family of evolutionarily related transaminases with completely conserved (>20 amino acid residues) active sites^3^. The work described the application of ancestral sequence reconstruction (ASR) as a tool in biocatalyst development and indeed the proteins described herein represent a modern day transaminase (annotated as KES23360) and its evolutionarily related ancestral counterparts (named N16 and N43). However, the discovery that our transaminases were so compositionally similar and yet catalytically distinct was both unexpected and unexplainable based on the current paradigm. Despite high overall sequence identity (average of 61%), structural conservation (average RMSD of 0.46 Å) and identical catalytic centres, the biocatalysts exhibited notable differences in substrate preference and turnover efficiency, suggesting that the local environment of the active site was having little or no influence on substrate preference (Fig. 1 and table S1). Transaminases are among the most widely utilised biocatalysts^4–6^, with a mechanism that is broadly well-characterised and numerous engineering successes reported^7–9^, but there is little precedent for our findings in the literature. As such, using these transaminases as exemplars, we designed a new methodology based on unbiased molecular dynamics (MD) simulations to try to rationalise our previous findings. The results described herein demonstrate that even in “well-characterised” protein families, the relationship between protein and substrate could be significantly more complex than conventionally understood, and when it comes to engineering proteins we are really only scratching the surface.

## Results and Discussion

Based on our previous experimental findings, we initially designed MD simulations to compare differences in protein dynamics between the transaminases. Each of the three proteins was studied both with and without a bound substrate molecule, but no significant differences were observed. We hypothesised that, if the residues comprising the active sites were conserved, and the proteins exhibited no discernable dynamic differences *in silico*, the differences in catalytic properties must be a consequence of the way that substrate molecules reach the active site in each protein. Substrate trajectories must therefore be influenced by the exterior surface of the proteins, with surface-directed ligand diffusion providing an important contribution to catalysis. However, in contrast to the active sites, the protein surfaces contained many amino acid variations, and identifying the residues involved in catalysis was not facile. As mentioned at the outset, this complexity is why most rational engineering strategies, with the exception of those aiming to improve thermostability or halophilicity, overlook the surface. As such, we devised a new MD approach to analyse protein-substrate interactions and attempt to identify the regions on the surface of the proteins which contribute to substrate selectivity and catalysis. This differs fundamentally from enchanced sampling techniques^2^ that have been employed to date, such as steered molecular dynamics or locally enhanced sampling in that it uses a wholly canonical, unbiased MD approach to generate multiple parallel trajectories, followed by application of a novel clustering algorithm to reduce the resulting complexity.

**Figure 2:**
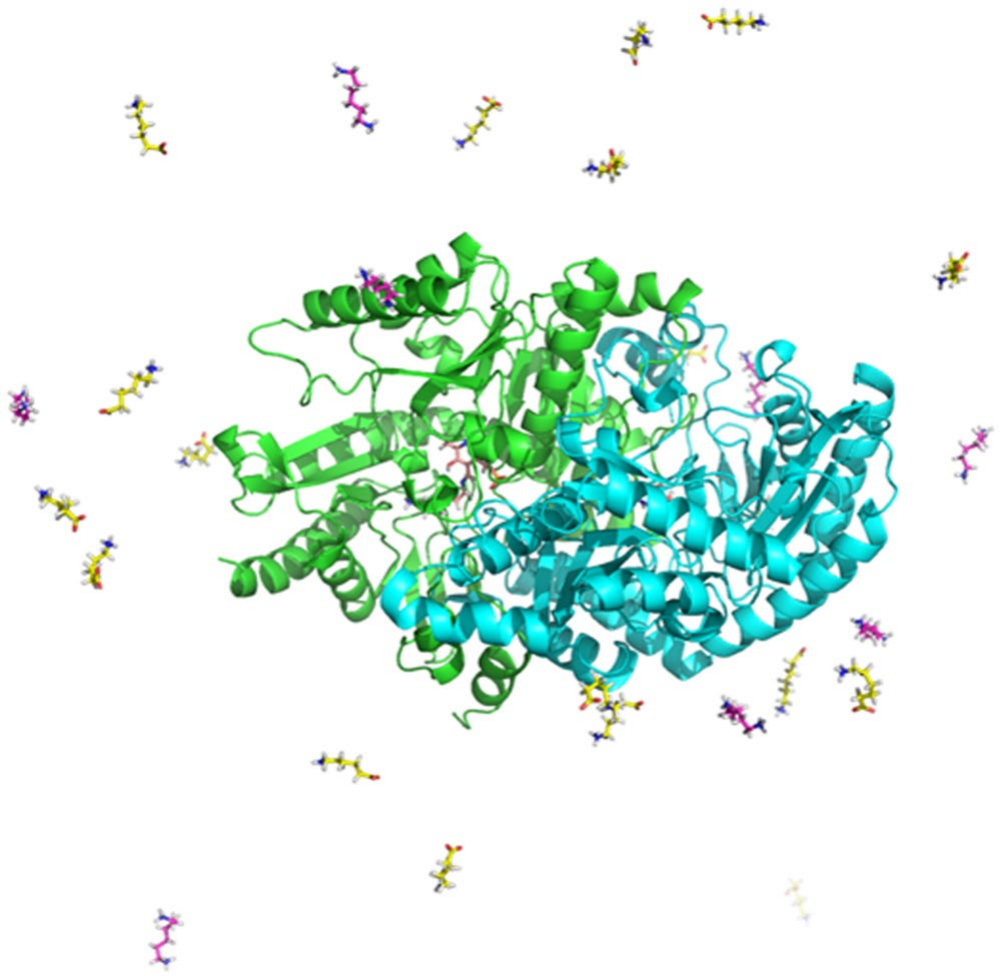
Single frame snapshot from one of the MD simulations. The KES23360 dimer is shown in cartoon representation with cofactors within the active sites coloured orange, and the substrates in solution coloured yellow (AHA) and magenta (HMD). Solvent has been removed for clarity.

The new approach started from dimeric transaminases with one active site containing the pyridoxal-5′-phosphate cofactor (PLP; the “empty” active site) and one containing PLP bound to a molecule of 1,6-hexamethylenediamine (HMD) in an external aldimine conformation (the “occupied” active site; consistent with our previous protein dynamics studies). In the new approach, additional substrate molecules were added to the system at random positions in solution around the protein. Further, multiple copies of two different substrates were added to increase the likelihood of observing differences; twenty molecules of each of 6-aminohexanoic acid (AHA) and HMD were added to the solution *in silico* (to a final concentration of approximately 15 mM; Fig. 2). These substrates were selected because the transaminases had all previously exhibited a preference towards the ω-amino acid compound experimentally, but to different degrees^9^. They were also of interest because they have applications in industrial polyamide synthesis, and finally, the choice allowed a comparison of substrates with different charge properties but minimal steric variation.

**Figure 3:**
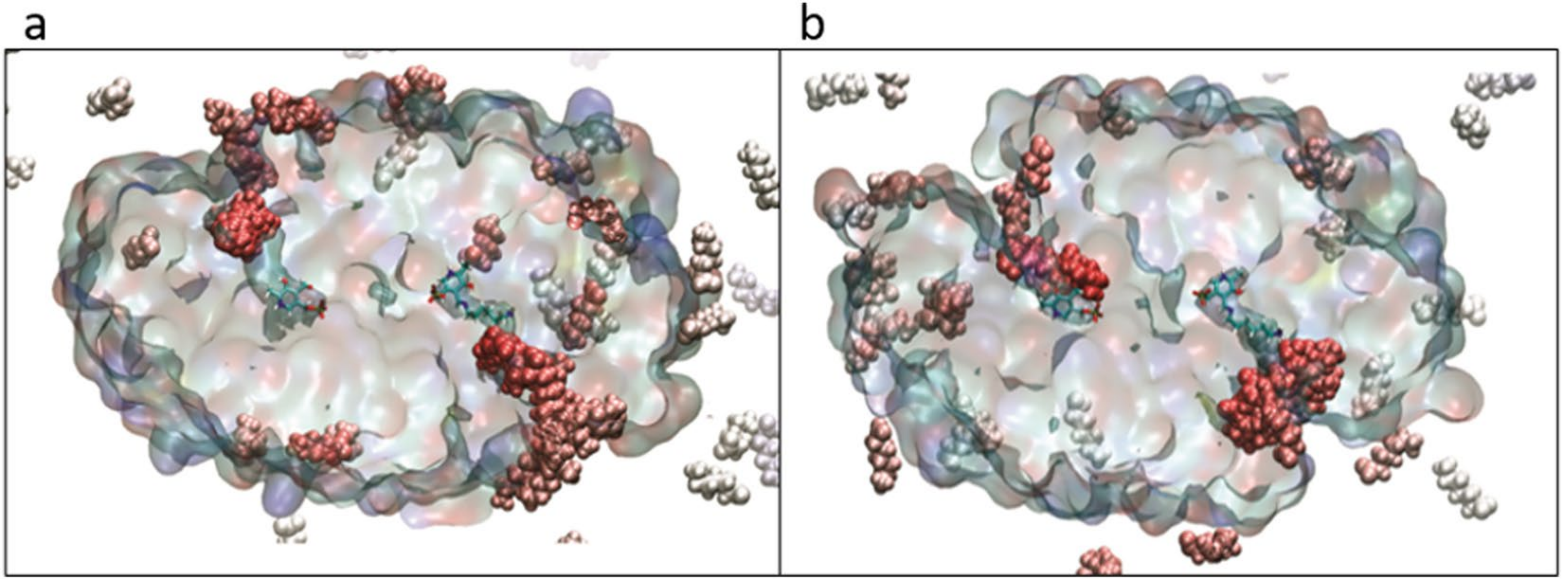
A representation of the *in silico* substrate competition assays. Overlay of frames for the substrates taken over 25  ns. Cross sections of the dimeric KES23360 (panel a) and N43 (panel b) proteins are shown as a surface, with cofactors in unbound and bound forms (PLP and the HMD:PLP aldimine complex, respectively) shown in a single conformation and in stick representation. AHA molecules are shown as spheres and coloured using a blue/white/red colour scale, relative to their proximity to the centre of the protein. The AHA molecules clearly localise in the active site of the protein in proximity to the cofactors.

Initially, MD simulations were performed in triplicate on the three transaminase systems and after 200 ns, the nine simulations were compared. Interestingly, we found that there was a significant difference in the way that the two substrates interacted with the proteins. The AHA molecules were observed to interact broadly over the surface of the proteins, while the diamines made only superficial interactions and only associated on very specific regions of the protein surface. We also found that, in several cases, AHA molecules had travelled from bulk solvent into the active sites of the enzymes, within hydrogen bonding distance of the PLP cofactor (Fig. 3). This was an unexpected finding given the timeframe of these initial simulations, and relying solely upon diffusion with no additional steering force imposed, but it was observed in six of the nine simulations. With development, this type of approach could potentially serve as a primitive *in silico* substrate screening assay for biocatalysts in the future. Encouraged by this result, we expanded the study and increased the rigor of the experiment, repeating simulations for each of the three systems thirty times to give ninety 100 ns simulations for a total of 3 μs total simulation time per protein system (~120,000 atoms per system, total of 35,000 CPU hours and 35,000 GPU hours).

**Figure 4:**
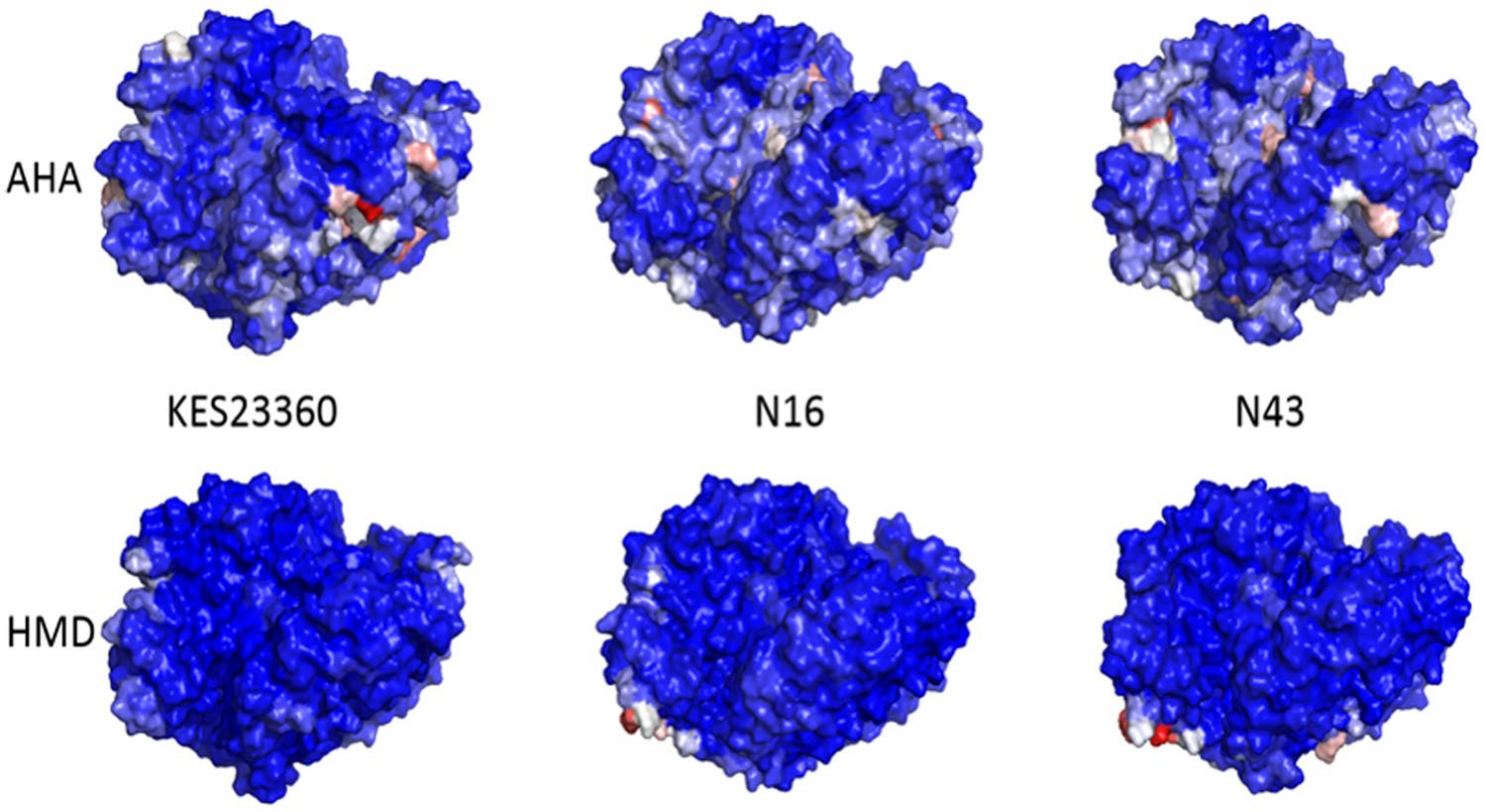
RDF Analysis comparing AHA (top) and HMD (bottom) with the three protein systems. Protein surfaces are coloured using a blue-white-red colour scale with red representing a high RDF score. AHA molecules clearly associated with the protein surfaces more than HMD, and there were clear differences in AHA association loci between the proteins.

After the ninety simulations had each been run for 100 ns, the trajectories were analysed and, consistent with previous results, we found that AHA molecules travelled into the active sites of the transaminases in over 50% of the simulations (16, 15 and 15 in the ‘empty’ active sites and 25, 22 and 21 in the ‘occupied’ active sites for KES23360, N16 and N43, respectively). In the ‘empty’ active sites AHA molecules were often observed within hydrogen bonding distance of the PLP aldehyde in a biochemically relevant conformation, and further, retained for the duration of the simulation thereafter. In contrast, although slightly higher occupancy was observed in the already occupied active sites, since these sites already contained substrate, penetration into the active site cavity from AHA molecules was always comparatively superficial. Importantly, HMD molecules were not observed in either active site in any of the ninety simulations. Next, to elucidate how the AHA molecules travelled to the active site, we sought to identify the surface residues with which they interacted or even determine if substrate channels existed on the surface of the protein. We first used radial distribution function (RDF) analysis, which provided a statistical measure of the substrate population at each amino acid residue for AHA summed over the course of each simulation. This score could then be averaged over the thirty simulations for each system; we visualised this data by writing the per-residue values to a pdb file and then colouring each residue by RDF score (Fig. 4). From these analyses, it was clear that AHA interacted with the protein much more frequently than HMD and that the substrates behaved differently with each protein, but reliably identifying the specific residues or regions of the protein surface using this methodology was impractical, if not impossible.

Instead, we decided to approach the problem from a different perspective and instead of comparing the protein surfaces, we analysed the molecular trajectories of the substrate molecules themselves. With 3,600 individual AHA and HMD trajectories to analyse, each consisting of ten thousand frames of data, visual inspection or conventional trajectory analysis tools were not sufficient for the task. To solve this problem, we developed an algorithm to reduce the data and extract meaningful information.

First, the atomic co-ordinates for each AHA or HMD molecule in each frame were converted to a single centroid (a spatially averaged xyz co-ordinate for the molecule) reducing the data approximately 25-fold. Next, the trajectories were individually analysed using a modified clustering algorithm. The clustering algorithm took the co-ordinates for each substrate centroid, for each of the 10,000 frames, for each trajectory, and found its nearest unique neighbour of the same type in 3D space. It then created a new centroid from the spatial average of the two points and exported this centroid to a new pdb file. This was repeated iteratively until all the co-ordinates had been reduced to a single point or handful of points. A distance cut-off of 10 Å was incorporated for each clustering iteration to remove isolated centroids in low-density regions and eliminate ‘false’ centroids that would otherwise be generated that did not represent properly co-located substrate instances. The overall effect was to filter the substrate down to a handful of the most populated positions in space. The centroid clusters could then be superposed onto the protein structure. An illustration of these sequential outputs and an overlay is shown in Supplementary Materials Figures S2 and S3.

**Figure 5:**
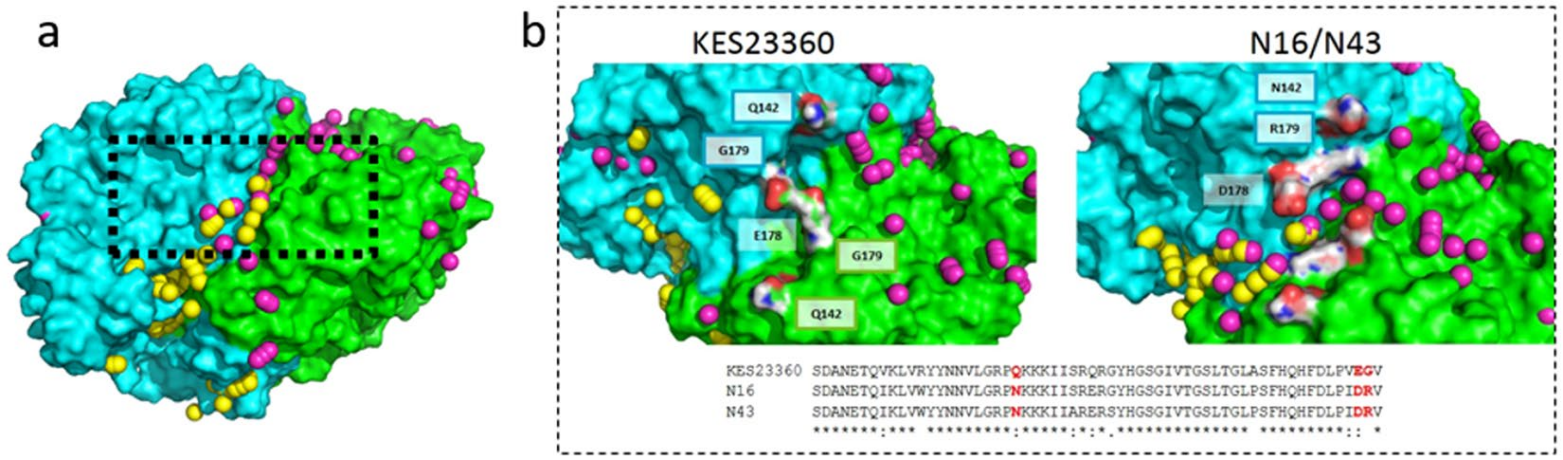
Cluster analysis performed on AHA molecules with from the N16 system. The centroids are coloured according to the active site they ultimately occupy, with those occupying the ‘empty’ site in yellow and the ‘occupied’ site in pink (panel a). The monomers comprising the protein dimer are coloured cyan and green and the boxed area highlights the region of the surface over which both trajectories converge. (Panel b) Closer analysis and comparison of the boxed region showing non-conserved residues that contribute to an open or closed channel through which substrate appears to move. In KES23360, E178 appears to dam the channel, whilst in N16/N43 the corresponding D178 does not. Other non-conserved residues in the region (positions 142 and 179) are highlighted in white with heteroatoms coloured (O – red, N – blue), and labelled accordingly. A sequence alignment of the region is also shown.

Combined analysis of all the AHA trajectories did not provide any further insights over the RDF data. However, analysing only the trajectories of the molecules which travelled to the active sites was very revealing. Whilst the KES23360 system remained unremarkable, analysis of the N16 and N43 systems showed that the clusters appeared to form paths along the surface of the proteins. Working back from the active sites, the trajectories both originated from the same region on the surface of the dimer, far away from the active sites, and furthermore this region on the surface was consistent in N16 and N43. AHA molecules appeared to diffuse to both active sites in opposite directions from this region, and further, at the sequence level the residues comprising this region were conserved between N16 and N43 (Fig. 5). To ensure that this was not an artefact of the approximation imposed by using centroids, we also went back to the original simulations and confirmed that substrate molecules arriving over this region of the protein surfaces did indeed follow the observed trajectories.

A comparison of the sequences, and even structures, in this region of the three proteins is unremarkable without context (Asp178 in N16/N43 is Glu in KES23360; Sequence alignment shown in Supplementary Figure S1), but with the trajectory data overlayed this comparison reveals a striking structural feature that distinguishes KES23360 from N16 and N43. Whilst the smaller aspartates contribute to the walls of a channel along the surface of the protein, the larger glutamates appear to bridge it, essentially forming a dam, and disrupting the *in silico* “flow” of substrate over the surface (Fig. 5). Other prominent amino acids in this region conserved in N16 and N43 include an arginine at position 179 (glycine in KES23360) and an asparagine at position 142 (glutamine in KES23360), and whilst the roles of these residues are less apparent we decided to empirically investigate the roles of these residues as well. We performed site-directed mutagenesis at positions 142, 178 and 179 in KES23360 to the N16/N43 equivalent residues, both individually and in combination, and determined pseudo one-substrate kinetics for AHA for each.

The experimentally derived kinetics (Table 1) showed that KES23360 marginally outperformed N16 and N43, but the KES23360 variants exhibited more significant improvements (up to ≈4-fold) in catalytic activity towards AHA. Although the contribution of each residue is difficult to elucidate, the data supports the overall findings of our MD study and suggests that these residues contribute to a substrate channel that did not previously exist in KES23360. However, considering that N16 and N43 are both ancestral peptides, which are generally considered to be more versatile but lower efficiency catalysts than their modern day counterparts, it is perhaps unsurprising that their kinetics can be further improved by sampling point mutations. Diffusion from bulk solvent appears to be the primary route for AHA into KES23360, but we would suggest that the secondary channel installed in the variants appears to both sequester and supply AHA from bulk solvent to both active sites and likely results in the catalytic improvements we observed. To our knowledge this is the first prediction, rational design, installation and experimental validation of a surface substrate channel into any protein. These findings also demonstrate that biocatalysts can be further improved by engineering substrate channels into the surfaces of enzymes, and opens up an exciting new area for biocatalyst design. This is the first evidence of surface-directed ligand diffusion in transaminases, as well as the first experimentally supported engineering of this type. Importantly, these investigations illustrate that there are still gaps in our understanding, even in well-characterised protein families.

**Table 1:** Pseudo one-substrate kinetics calculated for AHA with each of the three transaminases and KES23360 variants.

Variant	*k*_cat_/*K*_M_ (M^−1^.s^−1^)
KES23360	88.6 ± 2.6
N16	66.1 ± 3.6
N43	52.5 ± 3.5
**KES23360 Variants**	
E178D	339.7 ± 27.2
G179R	330.2 ± 25.9
Q142N	153.7 ± 9.5
E178D G179R	145.3 ± 13.9
E178D Q142N	218.7 ± 10.9
G179R Q142N	274.2 ± 15.8
E178D G179R Q142N	176.7 ± 8.7

Specificity constants were calculated using an alanine dehydrogenase coupled assay as described in the Methods section, keeping the concentration of pyruvate constant and varying the concentration of AHA. All the KES23360 variants displayed improved kinetic properties.

In conclusion, we have described and validated a new computational approach, and developed new tools to probe the structure-function relationship of enzymes. While the concept of surface-directed diffusion of substrates has been proposed before^10,11^, only coarse-grained cellular automata models have since been developed^12^. Recently, progress has been made in the identification of ligand binding ‘hotspots’ using Adaptive Biasing Force (ABF) techniques in molecular dynamics simulations^13^, which has some advantages over other commonly used methods typically aimed at examining direct pathways from bulk solvent to active sites, such as Steered Molecular Dynamics (SMD). However, applied to a well-characterised protein family, in this case the transaminases, we have for the first time simulated substrate diffusion over the surface of the protein from first principles using atomistic simulations and no biasing force. The notion of “surface channels” identified by our simulations was further experimentally supported by the rational design of surface variants with improved catalytic properties, suggesting that these channels not only exist, but contribute to catalytic efficiency as predicted *in silico*. As stated previously, this is to our knowledge, the first example of biocatalyst engineering in this manner, but it is likely that we are only “scratching the surface” in this field. As described at the outset, the role of the surface of proteins is understudied and underutilised, in part because tools to analyse and interpret the data have until now not existed. Given there is nothing overtly remarkable about transaminase proteins or the substrate used in this study, we have no immediate reason to expect that the applicability of the methods described would be limited to this system. Theoretically, the tools and approach presented here could be applied to any protein, and used to introduce new channels into surfaces for improved performance.

## Methods

### Protein Production

Protein overexpression was achieved by growing 500 mL of *E. coli* BL21 λDE3 containing the desired vector in LB media containing ampicillin (100 μg/mL) at 37 °C. When the OD_600_ reached 0.6–1.0, the cultures were induced by the addition of IPTG (Isopropyl β-D-1-thiogalactopyranoside; 1 mM final concentration) and further incubated at 15 °C for 18 hours. The cells were isolated by centrifugation (4000 × *g*; 20 minutes) and the supernatant discarded. The pellet was resuspended in imidazole/sodium chloride/potassium phosphate buffer (5 mM/500 mM/10 mM, pH 7.5) and cell lysis was achieved using an Avestin C3 Emulsiflex Homogeniser at 20 kpsi. Cellular debris was pelleted by centrifugation (40,000 × *g*, 45 minutes) and the supernatant was passed over a HiTrap Chelating HP column (GE Healthcare) on an Åkta FPLC (Fast Protein Liquid Chromatography, GE Healthcare). Protein was eluted with an increasing concentration of imidazole (5–500 mM) and the separated protein was transferred into potassium phosphate buffer (10 mM, pH 7.5), concentrated by centrifugation (GE Healthcare; 10 k MWCO) and further purified by gel filtration (Superdex 200; G.E. Healthcare) in the same phosphate buffer. Purity for all proteins was estimated to be >95% by SDS-PAGE.

### Activity Assays

Transaminases were assayed using previously described methods^14^. Activities for each of the transaminases were assessed using enzyme-coupled dehydrogenase assays. A typical assay comprised: 6.25 mM substrate, 0.5 mM pyruvate, 1.25 mM nicotinamide adenine dinucleotide (NAD^+^), 0.035 U of alanine dehydrogenase (ADH; where 1 U corresponds to the amount of enzyme which converts 1 μmol L-alanine per minute at pH 10.0 and 30 °C), 2–50 nM transaminase, potassium phosphate (100 mM, pH 10). The catalytic rates of the transaminases were inferred from the coupled rate of NAD^+^ turnover by alanine dehydrogenase, which was dependent on the production of co-product (alanine) by the transaminase. NAD^+^ turnover was measured by the change in UV absorbance at 340 nm using a SpectraMax M2 spectrophotometer (Molecular Devices, Australia); reactions were conducted at 28 °C. Kinetic parameters were obtained using the above method, recording initial rates of activity across a range of substrate concentrations. Parameters were subsequently calculated using non-linear regression.

### Molecular Dynamics (MD)

Homology models of KES23360, N16 and N43 were prepared in Accelrys Discovery Studio v3.5 using the experimentally derived structures as a template. Models for the HMD:PLP external aldimine intermediate and PLP (for N43) were created in Accelrys Discovery Studio v3.5 and relaxed using the Full Minimization tool in Discovery Studio v 3.5 using the default settings (CHARMm forcefield). HMD-PLP aldimine complexes were manually orientated in the active site using the electron density from native PLP as a guide. For N43, no PLP was solved in the initial X-ray structure, so this was constructed *in silico* and manually orientated based on alignment against KES23360 and N16. Atomic charges were calculated in Accelrys Materials Studio v8.0 using the QEq method and the substrates were manually docked into the active site. Ligands were prepared for MD using the Antechamber module, employing the Mulliken charge method in AMBER16^15^ and using the GAFF2 forcefield. The protein models were prepared for MD simulations using xLeap applying the ff14SB forcefield and charge-neutralised by the addition of Na^+^ ions. The proteins were solvated in a TIP3P truncated octahedral solvent box with a minimum 12 Å periodic boundary distance from the solute.

Initial minimisation of both systems was performed using AMBER16 over 10,000 steps under a constant pressure of 1 bar (Berendsen barostat). MD simulations of 200 ns with a step-size of 0.002 ps were performed at 310 K and 1 bar pressure with a 2 ps relaxation time. Bonds lengths on bonds involving hydrogen were constrained using SHAKE, and force evaluation on these bonds was not performed. Long range electrostatic interactions beyond 12 Å were treated with the particle mesh Ewald method. Trajectories were analysed using VMD (v. 1.9.2)^16^, cpptraj and in-house python scripts. Analysis was conducted on the final 150 ns of the simulation, removing the first 50 ns to ensure the systems had equilibrated as determined through RMSD analysis.

Substrate competition builds were constructed using the same systems as above. Models of twenty free HMD molecules and twenty AHA molecules were randomly positioned around each protein with the atomic co-ordinates of the substrates kept consistent for the three systems to minimise the chance of biasing a system towards a specific substrate. The systems were charge neutralised by the addition of Cl^−^ ions and solvated as above. Minimisation and production were performed as described above with production runs taking course over 100 ns. Simulations for each protein were carried out thirty times (ninety simulations total). Radial distribution functions for substrates for each of the protein residues were calculated using cpptraj in Amber16.

The workflow for the clustering calculations was as follows: Using cpptraj, each trajectory frame for a given trajectory was centered around residues 138–140. RMSD calculations (using the rmsd command in cpptraj) were performed using the first frame as a reference, and the trajectory was then re-centered using every protein residue. The protein, water and counterions were stripped and a single pdb file was generated containing all substrate atomic co-ordinates for each frame of the trajectory (400,000 substrates in total per trajectory). A centroid was generated for each substrate using an in-house python script and a new file written with each substrate now represented by a single x, y, z co-ordinate. The clustering algorithm was implemented using python and worked as follows: a list of each inter-centroid distance that was less than 10 Å was created, which was then reduced to a new list of centroids that was generated from each unique centroid pair, beginning with the pair having the shortest inter-centroid distance and ending with the pair having the inter-centroid distance closest to 10 Å. This process was repeated iteratively on the successively smaller lists until a single or small number of centroids remained. Centroid pairs with distances greater than 10 Å were removed from the list at the beginning of each iteration. A pdb file containing the centroids was generated at each of the iterations for overlay with the protein and visualisation in VMD.

## Electronic supplementary material

Supplementary Information

## Data Availability

Molecular dynamics trajectory files and analysis scripts can be provided upon request.
