# Supplementary material for: Computer-Guided Surface Engineering for Enzyme Improvement
Source: Sci Rep. 2018 Aug 10;8:11998. doi: 10.1038/s41598-018-30434-5 (PMC6086876; doi:10.1038/s41598-018-30434-5)
Supplement: Supplementary file 1 — Supplementary Information [file 41598_2018_30434_MOESM1_ESM.docx]

**Computer-Guided Surface Engineering for Enzyme Improvement**

Matthew Wilding, Colin Scott and Andrew C. Warden^*^

Supplementary Materials

**PDB IDs:**

5KQT (KES23360), 5KQU (N6), 5KQW (N15), 5KR3 (N16), 5KR4 (N17), 5KR5 (N43), 5KR6 (N48)

**Figure S1**: Alignment of the seven proteins. Master alignment attached separately in FASTA format.

Alignment performed in CLUSTAL O (v. 1.2.1).

KES23360 MTDYAKLFEQDRAHFMHPSTHAHDHASGALPGRIITGASGVRIRDHQGRELLDAFAGLYC

N6 MTDFDQLFEQDRAHFMHPSTHAHDHASGALPGRIITGASGIRIRDHEGRELIDAFAGLYC

N15 MTDLDQLFEMDRAHFMHPSTHAHDHASGALPGRIITGGKGIRIQDHEGREYIDAFAGLYC

N48 MTDTDDLLEMDRAHFFHPSTHLRDHASGELPGRIITGGKGIRIQDSEGREYIDAFAGLYC

N16 MQSLDQLFEMDRAHFMHPSTHAHDHASGALPGRIITGGKGIRIEDHEGREYIDAFAGLYC

N17 MTSLDQLFEEDRAHFMHPSTHAHDHASGALPGKIVTGGKGIRIEDHQGREYIDAFAGLYC

N43 MTSLEQLLEMDRAHFMHPSTHAYDHASGALPGRIITGGKGIRIEDHEGREYIDAFAGLYC

* . .*:* *****:***** ***** ***:*:**..*:**.* :*** :********

KES23360 VNIGYGRLEVADAIHEQAKQLAYYHTYVGHASEAIIELSARIIRDWAPAGMKKVYYGLSG

N6 VNIGYGRTEVADAIYKQAKELAYYHTYVGHSTEAIIELSSRIIRDWAPAGMKKVYYGLSG

N15 VNIGYGRTEVADAIYEQAKELAYYHTYVGHSTEAIIELSSRIIRDWAPAGMKKVYYGMSG

N48 VNVGYGRTEIADAIYEQAKELAYYHTYVGHSNEPIIELSERIIREWAPAGMSKVYYGMSG

N16 VNIGYGRTEVADAIYEQAKQLAYYHTYVGHSTDAIIELSSRIIRDWAPAGMKKVYYGMSG

N17 VNIGYGRTEVADAIYEQAKQLAYYHTYVGHSTDAIIELSSRIIRDWAPAGMKKVYYGMSG

N43 VNIGYGREEVADAIYEQAKQLAYYHTYVGHSNDPVIELSSRIIEDWAPAGMKKVFYGMSG

**:**** *:****::***:**********:.: :**** ***.:******.**:**:**

KES23360 SDANETQVKLVRYYNNVLGRPQKKKIISRQRGYHGSGIVTGSLTGLASFHQHFDLPVEGV

N6 SDANETQIKLVRYYNNVLGRPQKKKIISRQRGYHGSGIMTGSLTGLPSFHQHFDLPVEGI

N15 SDANETQIKLVWYYNNVLGRPQKKKIISRQRGYHGSGIMTGSLTGLPSFHQHFDLPIERI

N48 SDANETQIKLVWYYNNVLGRPQKKKIISRQRGYHGSGIMTGSLTGLPAFHNHFDLPLEPI

N16 SDANETQIKLVWYYNNVLGRPNKKKIISRERGYHGSGIVTGSLTGLPSFHQHFDLPIDRV

N17 SDANETQIKIVWYYNNVLGRPNKKKIISRERGYHGSGIVTGSLTGLPSFHQHFDLPIDRV

N43 SDANETQIKLVWYYNNVLGRPNKKKIIARERSYHGSGIVTGSLTGLPSFHQHFDLPIDRV

*******:*:* *********:*****:*:*.******:******* :**:*****:: :

KES23360 KHTLCPHFYKAPAGMDEAAFVRHCAQELENLILAEGPDTVAAFIGEPVMGTGGIIVPPKG

N6 KHTVCPHWYKAPAGMDEAAFVRYCADELEKLILAEGPDTVAAFIGEPVMGTGGIIVPPKG

N15 KHTVCPHWYKAPAGMSEAQFVRYCADELEKLILAEGPDTVAAFIGEPVMGTGGIIPPPQG

N48 RHTTCPHYYRAPAGMSEAEFSRHCADELEKMILAEGPDTVAAFIGEPVMGTGGIVPPPEG

N16 KHTVCPHWYRAPAGMSEAQFVAYCVEELEKLIAREGADTIAAFIAEPVMGTGGIIPPPQG

N17 KHTVCPHWYKAPAGMSEAQFVAYCVEELEKLIAREGADTIAAFIAEPVMGTGGIIAPPQG

N43 KHTVCPHWYNAPPGMSEAQFVAYCVEELEKLIAREGADTIAAFIAEPVMGTGGIVPPPQG

:** ***:*.** **.** * :*.:***::* ** **:****.*********: **:*

KES23360 YWEAIQAVLAKYDVLLIADEVVCAFGRLGDKMGSQRHAMRPDLITTAKGLTSAYAPLSAV

N6 YWEAIQAVLNKYDVLLIADEVVCAFGRLGSKMGSQRYGMRPDLITTAKGLTSAYAPLSAV

N15 YWEAIQAVLNKYDILLIADEVVCGFGRLGSKMGSQHYGMKPDLITVAKGLTSAYAPLSGV

N48 YWEAIQAVLNKYDILLIADEVVCGFGRTGSMFGSHHYGMKPDLITVAKGLTSAYAPLSGV

N16 YWEAIQAVLRKHDILLIADEVVCGFGRLGSKMGSQHYGIKPDLITVAKGLTSAYAPLSGV

N17 YWEAIQAVLRKHDILLISDEVVCGFGRLGSKMGAQHYGIKPDLITVAKGLTSAYAPLSGV

N43 YWEAIQAVLRKHDILLIADEVVCGFGRLGSKTGSEHYGIKPDLITVAKGLTSAYAPLSAV

********* *:*:***:*****.*** *. *:.::.::*****.************.*

KES23360 IVGEKVWDVIDSASTREGAMGHGWTYSGHPICAAAALANLDILERENITANAADVGGYLN

N6 IVGEKVWDVIEKASQKEGAMGHGWTYSGHPICAAAALANLDILERENLTANAADVGAYLN

N15 IVGEKVWDVIEKGSQEHGPMGHGWTYSGHPICAAAALANLDILERENLTGNAADVGAYLQ

N48 IVGEKVWKVLEQGSDQYGPIGHGWTYSGHPICAAAALANLDIIERENLTGNAADTGAYFQ

N16 IVGEKVWDVIEKGSQEHGPMGHGWTYSGHPICAAAALANLDILERENLTGNAADVGAYLQ

N17 IVGEKVWDVIEKGSQEHGPMGHGWTYSGHPICAAAALANLDILERENLTGNAADVGAYLQ

N43 IVSEKVWDVIEKGSREHGVMGHGWTYSGHPVCAAAALANLDILERENLTGNAADVGAYLQ

**.****.*::..* . * :**********:***********:****:*.****.*.*::

KES23360 QQLRQAFEGHPLVGEVRGDGMLAALEFMADREARTPFDAALKVGPKVSAACLERGMIARA

N6 QRLRETFEGHPLVGEVRGDGMLAALEFMADREARTPFDPALKVGPKVSAACLEDGMIARA

N15 QRLRETFGGHPLVGEVRGVGMLAALEFMADKDARTPFDPALKVGPKVSAACLEDGMIARA

N48 QRMRETFGDHPLVGEVRGVGLMAALEFVADKDKRTRFDPSLKVGPRVSAACLEDGMIARA

N16 QRLHEAFGAHPLVGEVRGVGMLAALEFMADKDARTPFDPALKVGPKVSAAALEDGMIARA

N17 QRLHEAFGAHPLVGEVRGVGMLAALEFMADKDARTPFDPALKVGPKVSAAALEDGVIARA

N43 QRLHEAFGAHPLVGEVRGVGMLAALEFMADKGARTPFDPALKVSQKVAAAALEDGLIVRA

*:::::* ********* *::*****:**: ** ** :***. :*:**.** *:*.**

KES23360 MPHGDILGFAPPLVLSRAEADEVVGIAKAAVDAVAAEVL

N6 MPHGDILGFAPPLVLTRAEADEIVGIAKAAVDEVAGEVL

N15 MPHGDILGFAPPLVITRAEVDEIVGIAKQAVDEVADEVL

N48 MPHGDILGFAPPLVITRAEVDEIVDIAKQAVDAVADELV

N16 MPHGDILGFAPPLVTTRAEVDEIVGIAKQAVDEVADEVL

N17 MPHGDILGFAPPLVTTRAEVDEIVGIVKQAVDEVADEVL

N43 LPHGDILGFAPPLVTTRAEVDEIVAIAKEAFDEVADAVL

:************* :***.**:* *.* *.* ** ::

**Figure S2**: Sequential clustering of one AHA trajectory with KES23360. This 10,000 frame trajectory with an initial total of 200,000 AHA molecules was subjected to ten sequential clustering iterations until a single centroid remained. Each output pdb contains centroids of one colour and the images are overlayed to illustrate convergence between points.


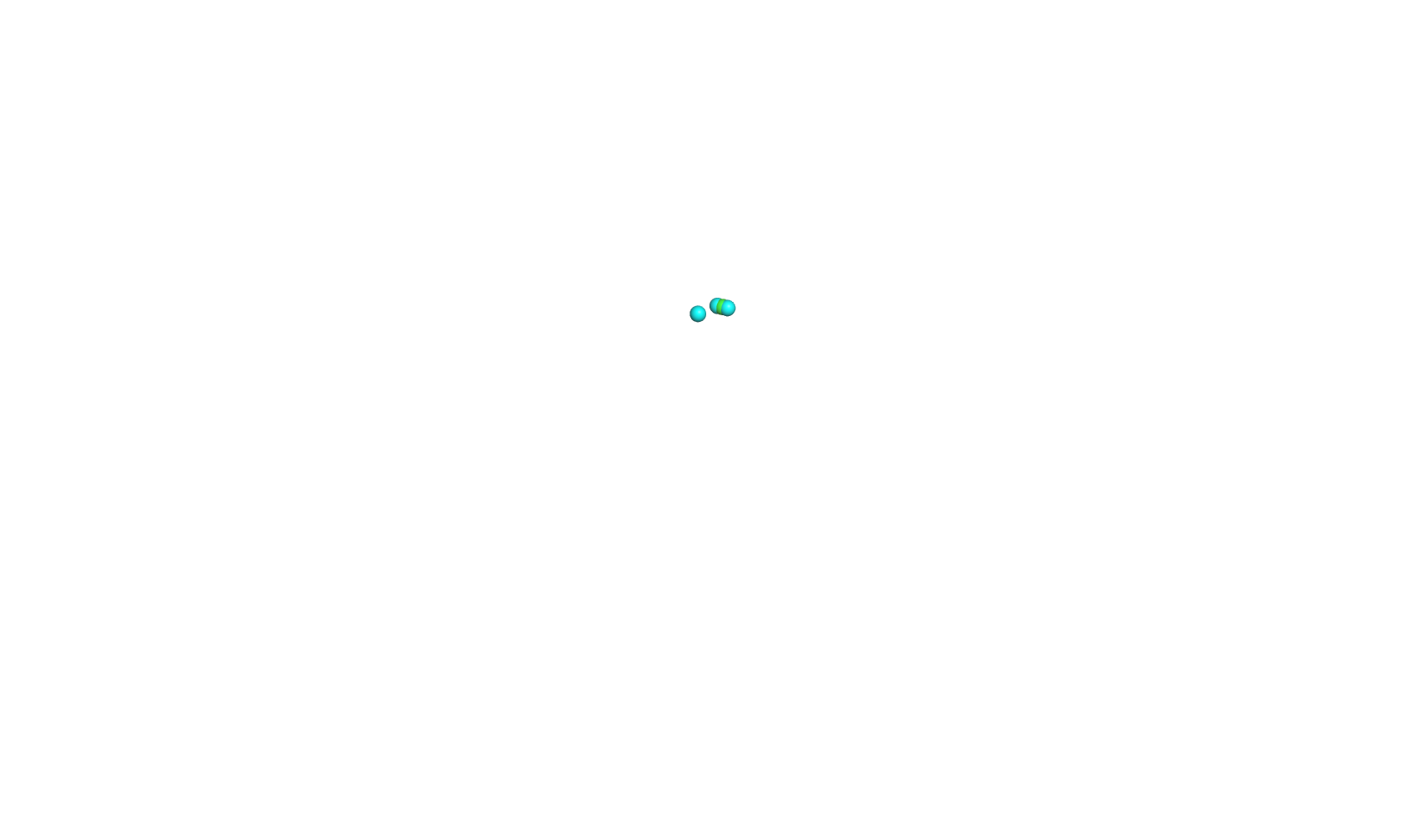

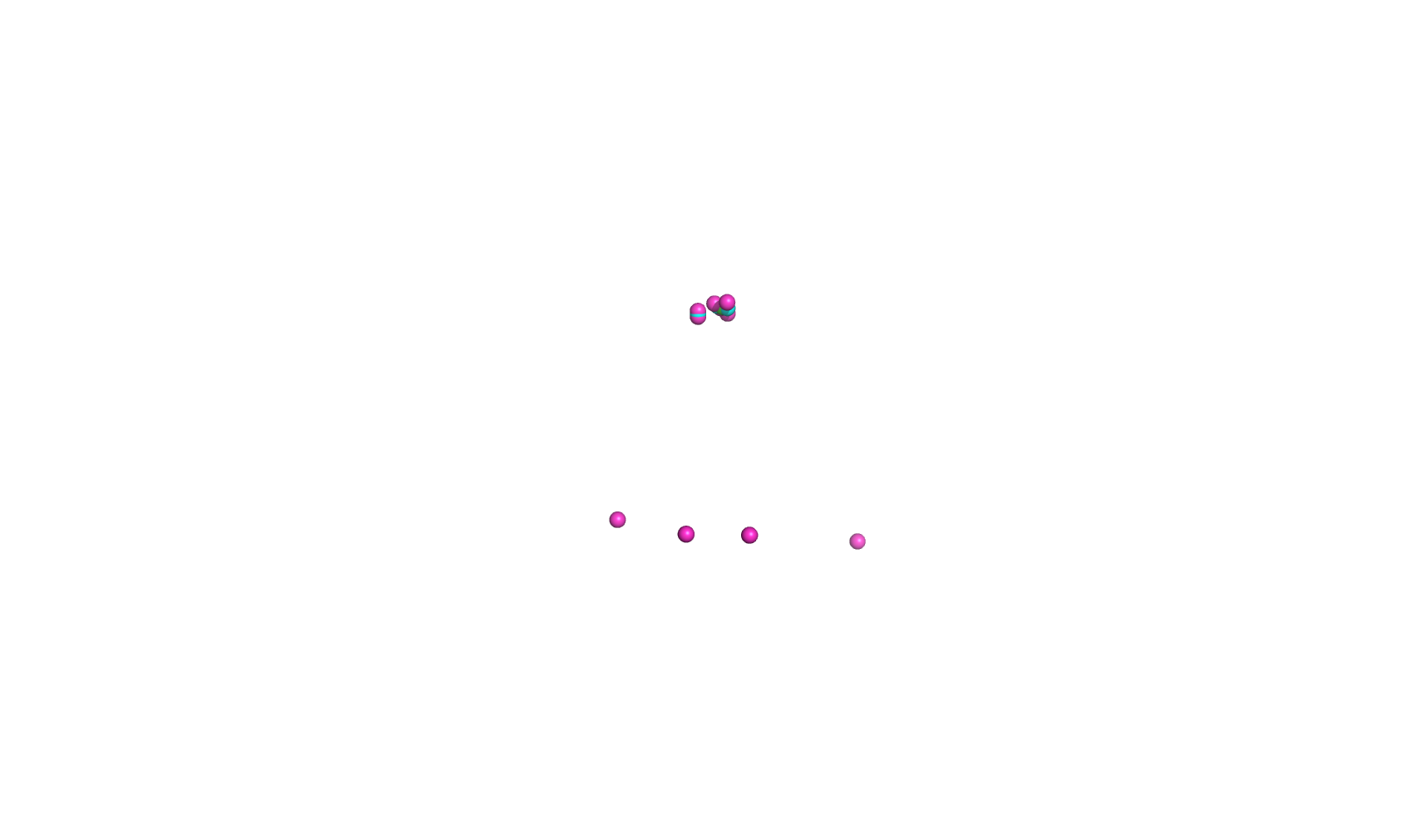

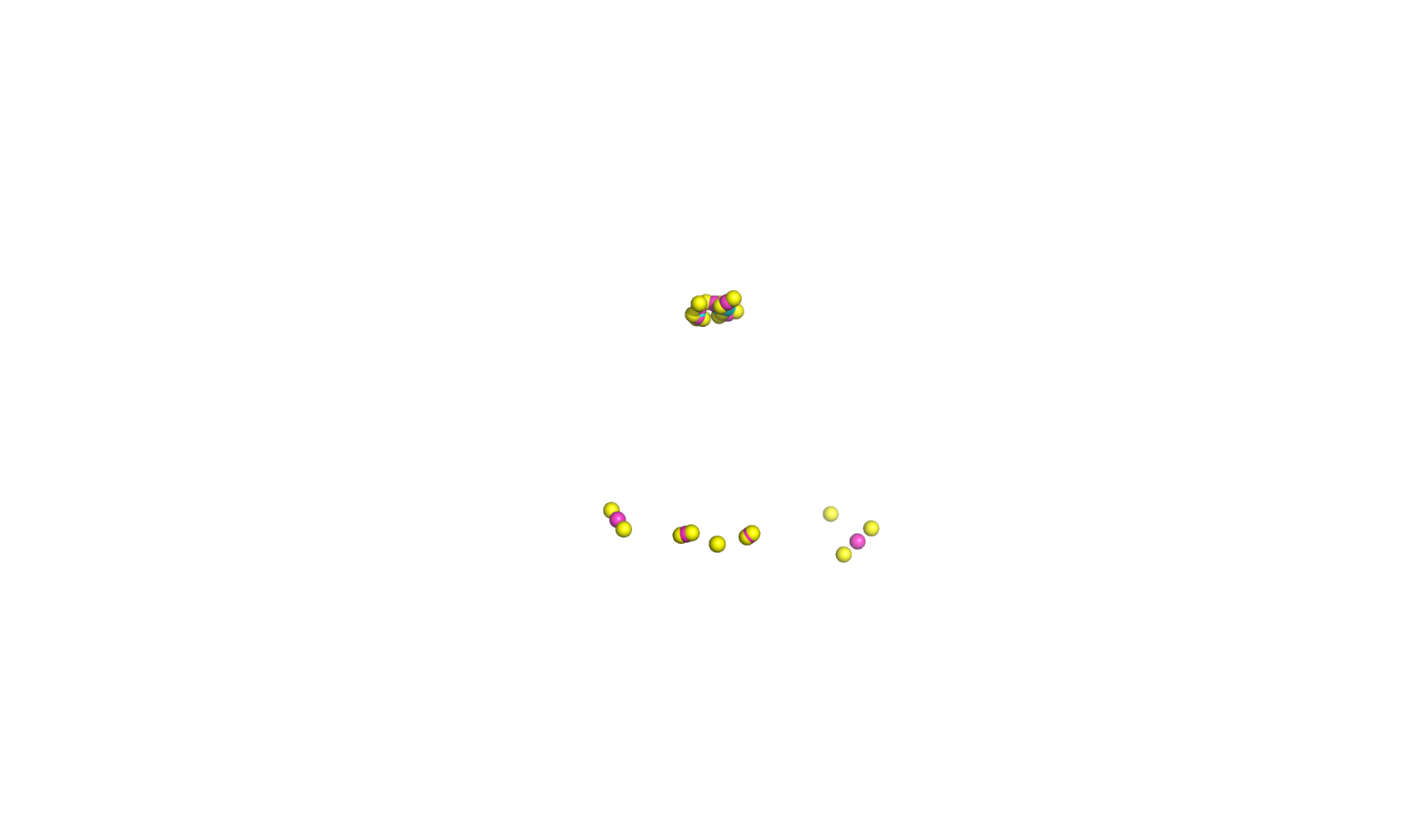

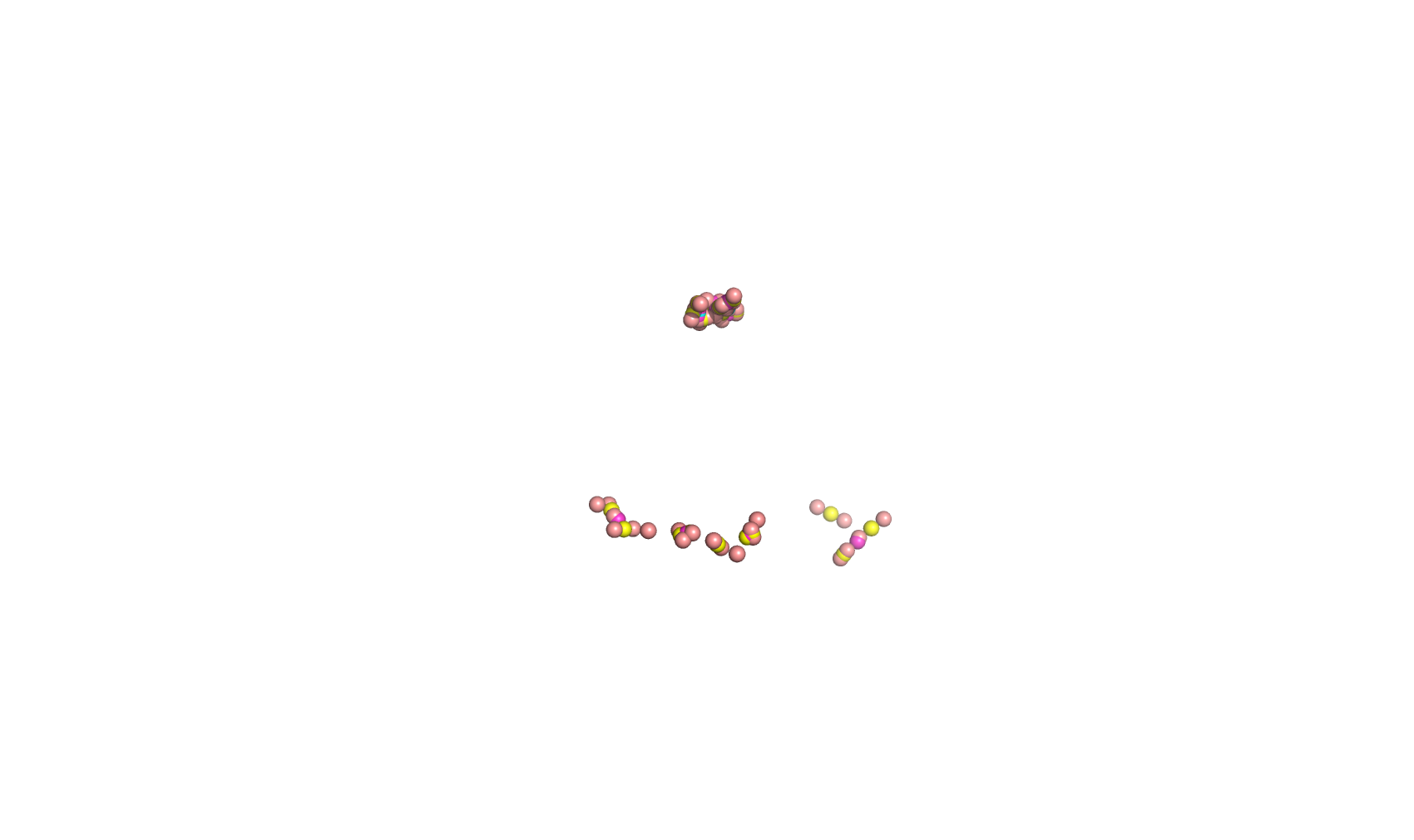

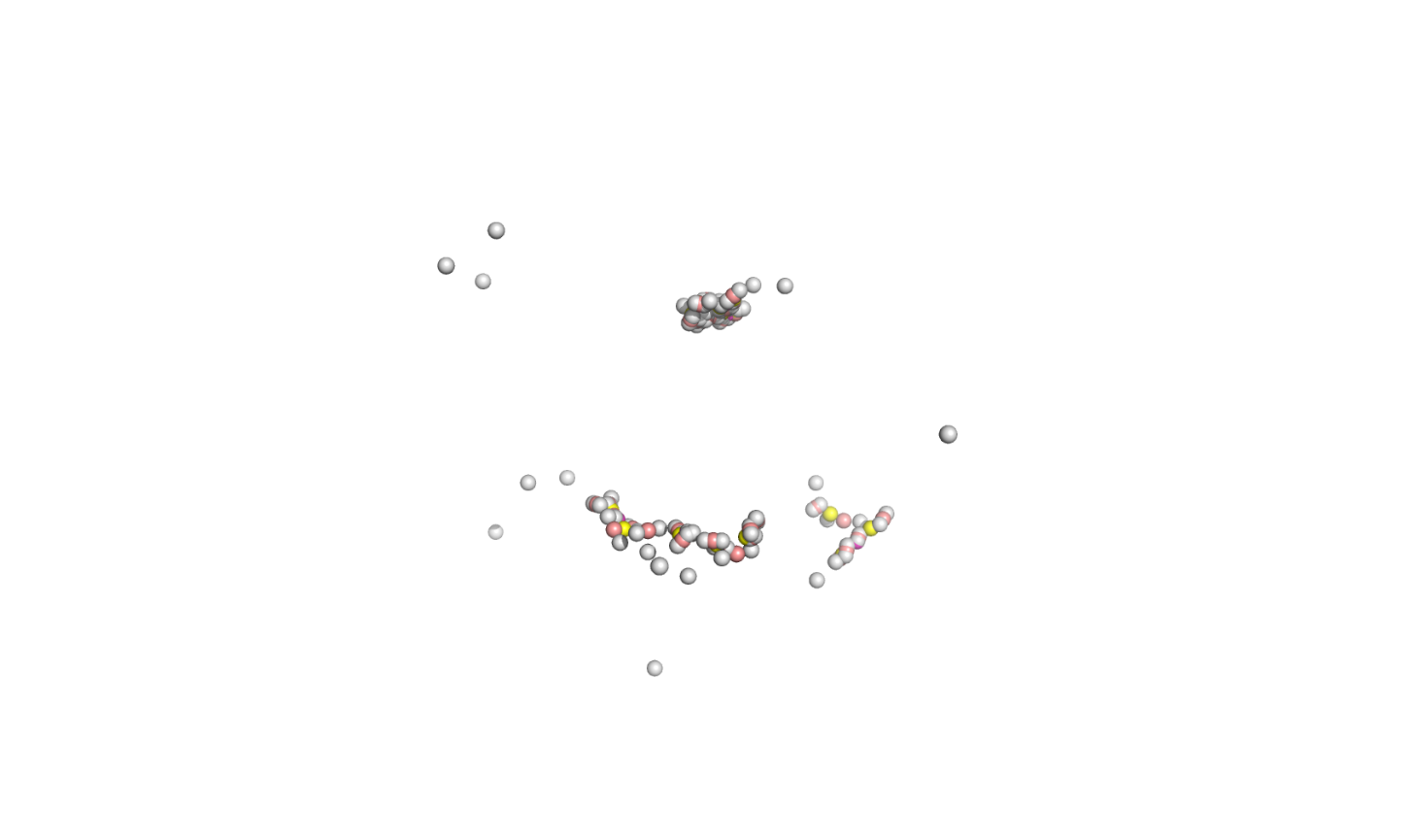

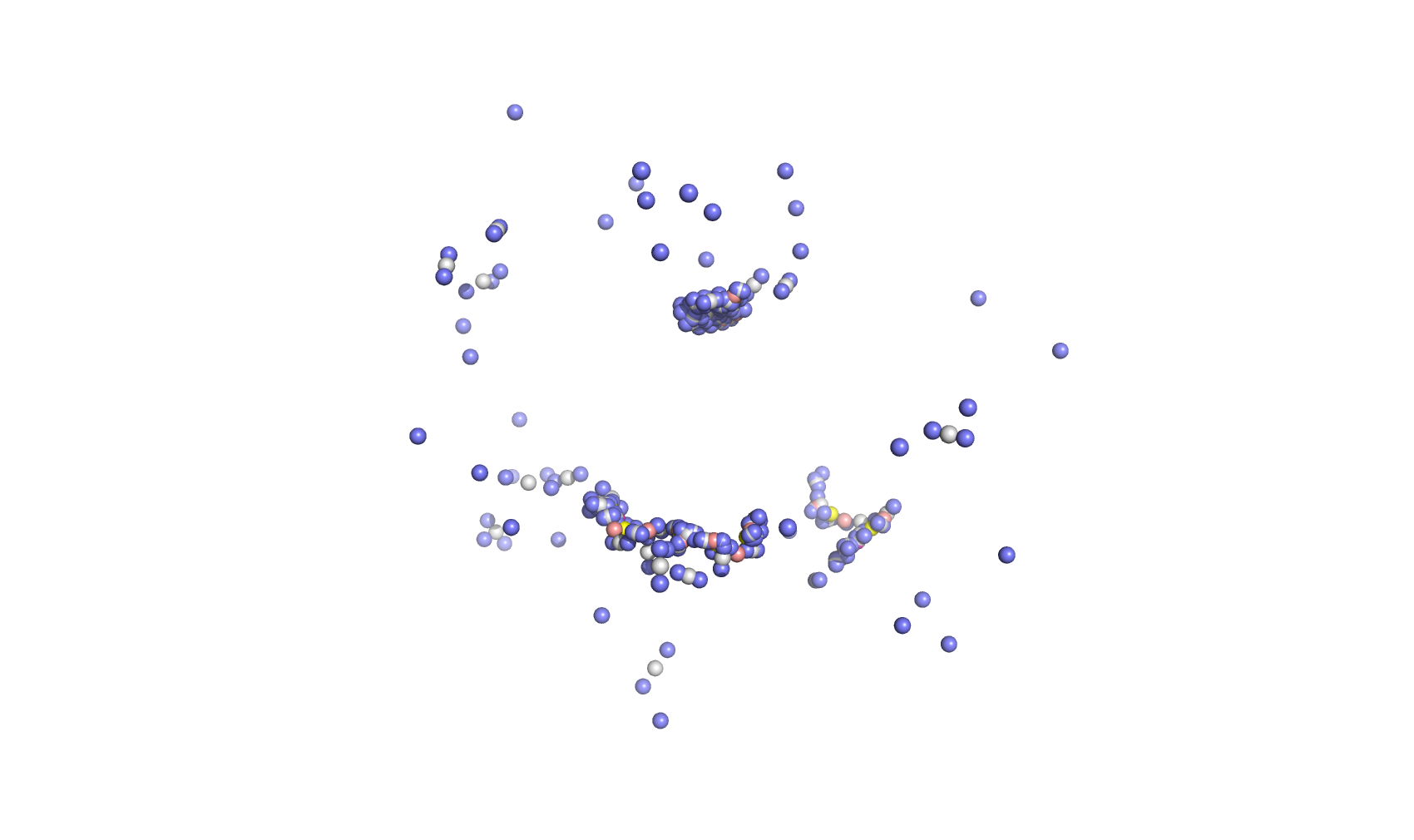

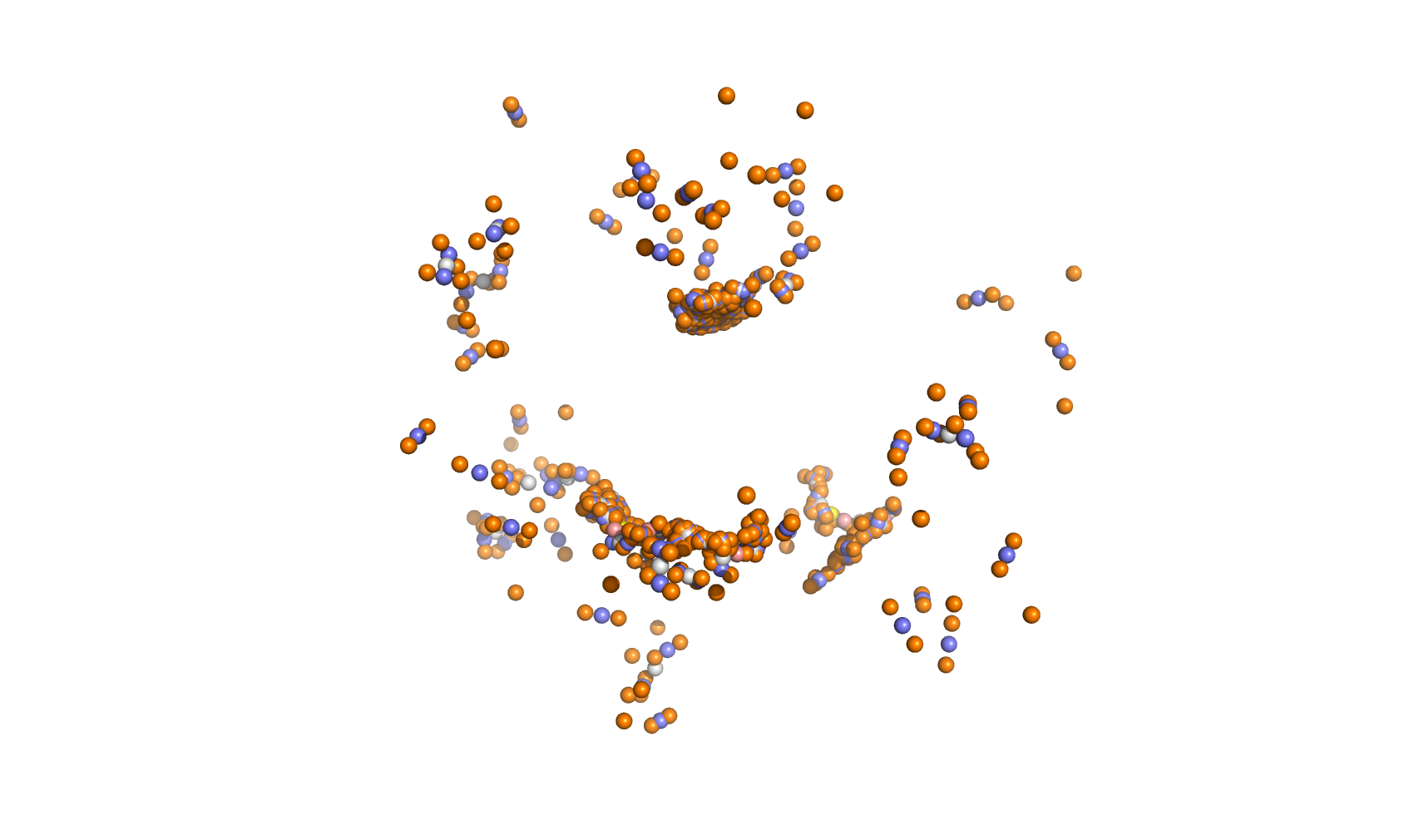

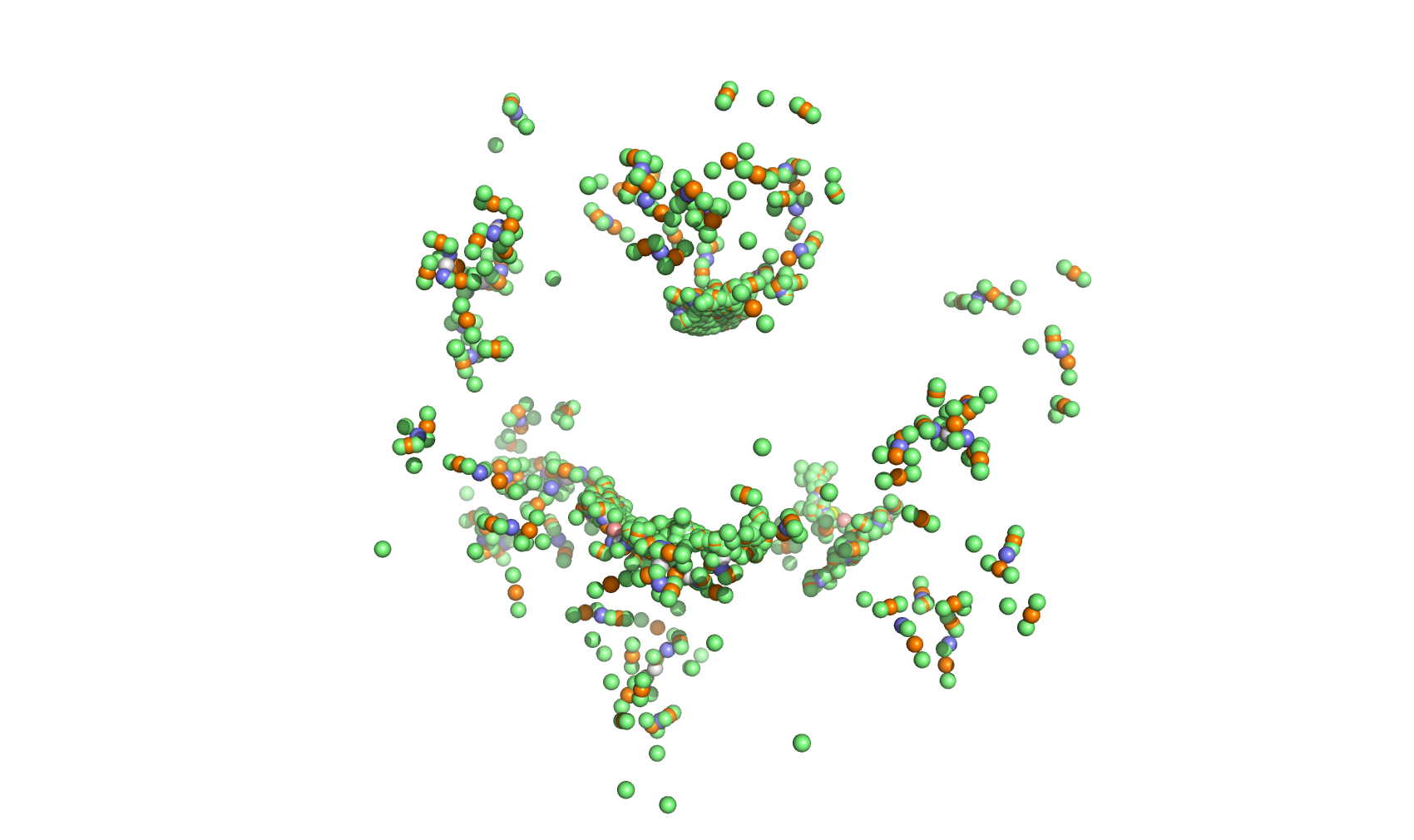

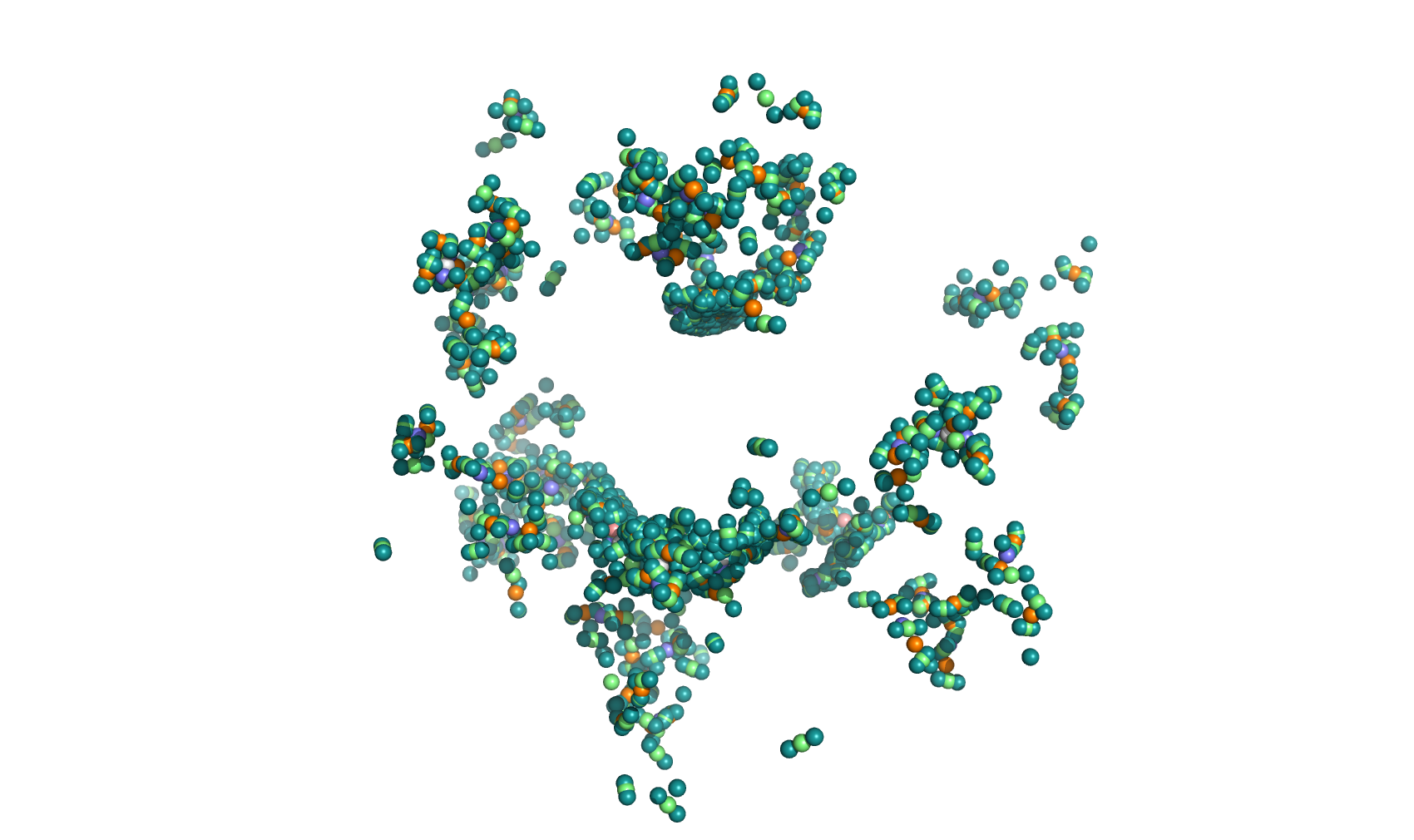

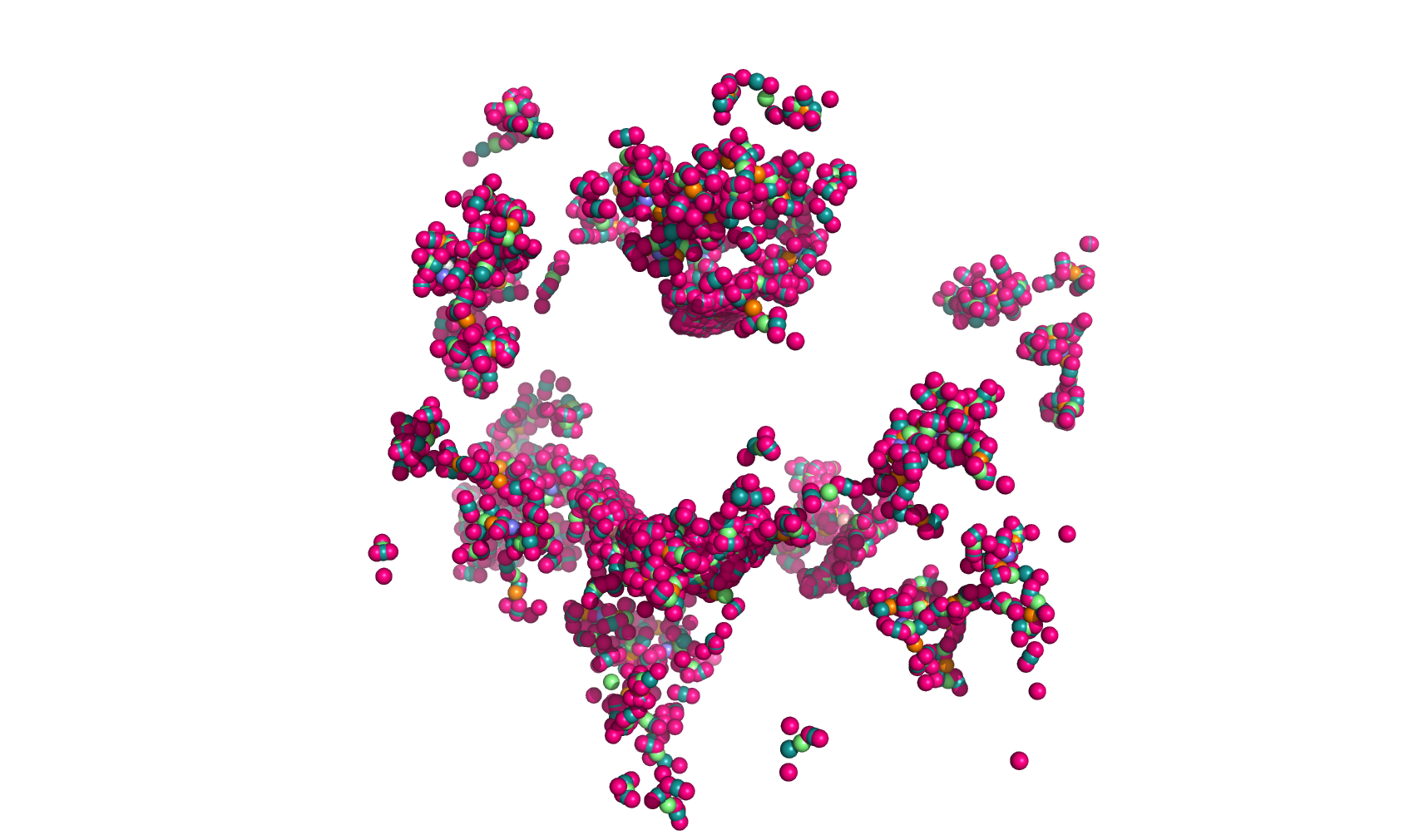

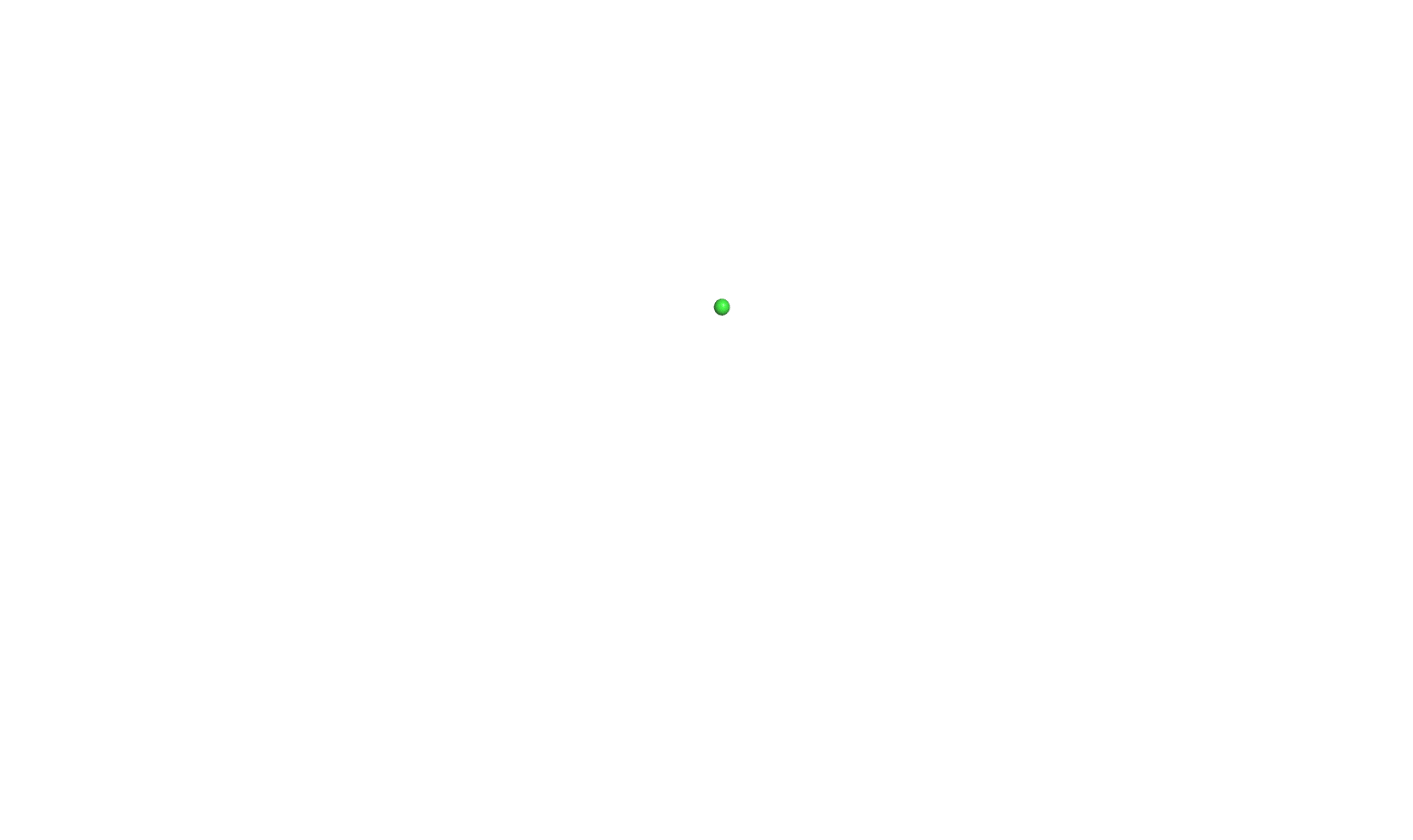


**Figure S3**: Superposition of the KES23360 protein against the original (10,000 centroids) and final (one centroid, green) outputs for one trajectory.

**Table S1**: Specific Activity and Relative Rate data for the ω-amino acids and α,ω-diamines with the tree transaminases. Specific activity data is presented in nmol/min/mg. Relative reactivity is shown in parentheses as a percentage, relative to the highest observed activity for each individual protein (treated as 100%).

| **Substrate** | **KES23360** | **N16** | **N43** |
| --- | --- | --- | --- |
| β-Alanine | 3.2 (3) | 0.4 (7) | 1.8 (2) |
| 4-Aminobutyrate | 48.1 (51) | 4.2 (77) | 49.7 (65) |
| 5-Aminopentanoate | 81.4 (86) | 5.5 (100) | 76.5 (100) |
| 6-Aminohexanoate (AHA) | 81.4 (86) | 4.5 (83) | 74.7 (98) |
| 7-Aminoheptanoate | 78.6 (83) | 4.2 (78) | 70.6 (92) |
| 8-Aminooctanoate | 64.9 (68) | 3.5 (64) | 61.0 (80) |
| 12-Aminododecanoate | 8.2 (9) | 0.6 (10) | 18.7 (24) |
| 4-NH_2_-(*S*)-2-hydroxybutyrate | 95.1 (100) | 4.6 (84) | 50.6 (66) |
| 2,4-(*S*)-Diaminobutyrate | 24.0 (25) | 1.5 (28) | 15.9 (21) |
| 1,3-Diaminopropane | 0.7 (1) | 0.1 (3) | 1.8 (2) |
| Putrescine | 15.1 (16) | 2.8 (51) | 32.3 (42) |
| Cadaverine | 4.7 (5) | 2.6 (47) | 62.0 (81) |
| 1,6-Hexamethylenediamine (HMD) | 5.4 (6) | 2.0 (36) | 37.8 (49) |
| 1,7-Heptamethylenediamine | 5.7 (6) | 2.1 (38) | 48.7 (64) |
| 1,8-Octamethylenediamine | 6.8 (7) | 1.7 (32) | 37.4 (49) |
| 1,9-Nonamethylenediamine | 12.2 (13) | 1.7 (31) | 43.7 (57) |
| 1,10-Decamethylenediamine | 7.2 (8) | 2.0 (36) | 38.3 (50) |
